# Hemostatic Factors and Risk of Coronary Heart Disease in General Populations: New Prospective Study and Updated Meta-Analyses

**DOI:** 10.1371/journal.pone.0055175

**Published:** 2013-02-07

**Authors:** Peter Willeit, Alexander Thompson, Thor Aspelund, Ann Rumley, Gudny Eiriksdottir, Gordon Lowe, Vilmundur Gudnason, Emanuele Di Angelantonio

**Affiliations:** 1 Department of Public Health and Primary Care, University of Cambridge, Cambridge, United Kingdom; 2 Roche Products Ltd., Welwyn Garden City, United Kingdom; 3 Icelandic Heart Association, Kopavogur, Iceland; 4 University of Iceland, Reykjavik, Iceland; 5 Cardiovascular and Medical Division, University of Glasgow, Glasgow, United Kingdom; Leibniz-Institute for Arteriosclerosis Research at the University Muenster, Germany

## Abstract

**Background:**

Activation of blood coagulation and fibrinolysis may be associated with increased risk of coronary heart disease. We aimed to assess associations of circulating tissue plasminogen activator (t-PA) antigen, D-dimer and von Willebrand factor (VWF) with coronary heart disease risk.

**Design:**

Prospective case-control study, systematic review and meta-analyses.

**Methods:**

Measurements were made in 1925 people who had a first-ever nonfatal myocardial infarction or died of coronary heart disease during follow-up (median 19.4 years) and in 3616 controls nested within the prospective population-based Reykjavik Study.

**Results:**

Age and sex-adjusted odds ratios for coronary heart disease per 1 standard deviation higher baseline level were 1.25 (1.18, 1.33) for t-PA antigen, 1.01 (0.95, 1.07) for D-dimer and 1.11 (1.05, 1.18) for VWF. After additional adjustment for conventional cardiovascular risk factors, corresponding odds ratios were 1.07 (0.99, 1.14) for t-PA antigen, 1.06 (1.00, 1.13) for D-dimer and 1.08 (1.02, 1.15) for VWF. When combined with the results from previous prospective studies in a random-effects meta-analysis, overall adjusted odds ratios were 1.13 (1.06, 1.21) for t-PA antigen (13 studies, 5494 cases), 1.23 (1.16, 1.32) with D-dimer (18 studies, 6799 cases) and 1.16 (1.10, 1.22) with VWF (15 studies, 6556 cases).

**Conclusions:**

Concentrations of t-PA antigen, D-dimer and VWF may be more modestly associated with first-ever CHD events than previously reported. More detailed analysis is required to clarify whether these markers are causal risk factors or simply correlates of coronary heart disease.

## Introduction

Because myocardial infarction usually results from occlusive thrombosis of atherosclerotic plaque, it has been proposed that circulating levels of markers that reflect activated coagulation and fibrinolysis may be associated with coronary heart disease (CHD) risk [1], [2]. Three such markers are tissue plasminogen activator (t-PA) antigen, D-dimer and von Willebrand factor (VWF). t-PA is the principal regulator of endogenous fibrinolysis catalyzing the conversion of plasminogen to plasmin [3]. In circulation, however, most of t-PA is bound to its inhibitor PAI-1 (plasminogen activator inhibitor type 1) and this complex can be measured as t-PA antigen [4], with high levels indicating biologically inactive t-PA and hence tendency to clot formation. D-dimer is a marker of activated coagulation and fibrinolysis and its levels reflect active fibrin mesh formation and degradation [5]. VWF is an important cofactor in platelet adhesion and platelet aggregation, key steps in primary hemostasis [6]. Previous studies have suggested significant and moderately strong associations of these markers with CHD risk in essentially general and secondary prevention populations [7]–[9], with perhaps stronger associations for myocardial infarction or fatal CHD than for incident stable angina, consistent with potential roles for activation of coagulation and fibrinolysis in coronary artery thrombosis, rather than atherogenesis [10]. To date, however, the available evidence for t-PA antigen and D-dimer has been limited to studies with only a few hundred CHD cases; insufficient to examine potentially important aspects such as the shape of dose-response relationships and the magnitude of these associations both overall and in clinically relevant subgroups with appropriate power.

To help clarify these uncertainties, we report new data on t-PA antigen and D-dimer involving 5541 participants in a prospective case-control comparison nested within the population-based Reykjavik Study of almost 19,000 middle aged Icelandic men and women monitored for a mean duration of about 20 years (results for VWF are presented here for comparison and have been reported previously [11], [12]). Context for our new data was provided by conducting a systematic review and updated meta-analyses of published reports on t-PA antigen, D-dimer and VWF and incident CHD.

## Methods

### Study Population

The Reykjavik Study, initiated in 1967, has been described in detail elsewhere [13]. All men born between 1907 and 1934 and all women born between 1908 and 1935 who were registered to live in Reykjavik, Iceland, or its adjacent communities on December 1^st^ 1966 were invited to participate in the study (**Figure S1**). During five stages of recruitment between 1967 and 1991, a total of 9139 men and 9773 women were enrolled (response rate = 72%). Nurses administered questionnaires, made physical measurements, recorded electrocardiograms and collected fasting venous blood samples for preparation of aliquots of serum, which were stored at –20°C for subsequent analysis. All participants were monitored by central registries for occurrence of major coronary events (based on MONICA criteria [14]) or cause-specific mortality (based on a death certificate with International Classification of Diseases), with a loss to follow-up of only about 0.6% to date. Cause-specific mortality was coded using ICD-9 and ICD-10. CHD deaths were pre-defined as those allocated ICD-9 codes 410–414 and ICD-10 codes I20–I25. We selected 1925 participants who suffered a first-ever non-fatal MI or coronary death between study entry and the censoring date (median duration of follow-up, 19.4 years) and without evidence of cardiovascular disease at baseline, in whom baseline serum was available for measurement of t-PA antigen, D-dimer and VWF. We selected a random subset of 3616 controls from among the participants who had survived to the end of the study period without developing CHD, frequency matched to cases by recruitment year, age (in five-year age bands) and sex. Baseline characteristics of selected controls were broadly comparable to those of the overall Reykjavik cohort (**Table S1**). The National Bioethics Committee and the Data Protection Authority of Iceland approved the study protocol. All participants gave informed written consent and the study complies with the Declaration of Helsinki.

### Laboratory Methods

All measurements were made in serum samples, by laboratory staff unaware of participants’ disease status. Enzyme immunoassays were used to determine levels of t-PA antigen (Biopool AB, Umea, Sweden), D-dimer (Hyphen, Paris, France) and VWF (in-house assay using reagents from DAKO, Copenhagen, Denmark). All intra- and inter-assay coefficients of variation were <5%. Lipid and other biochemical measurements have been described previously [11]–[13].

### Statistical Analyses

**Figure 1:**
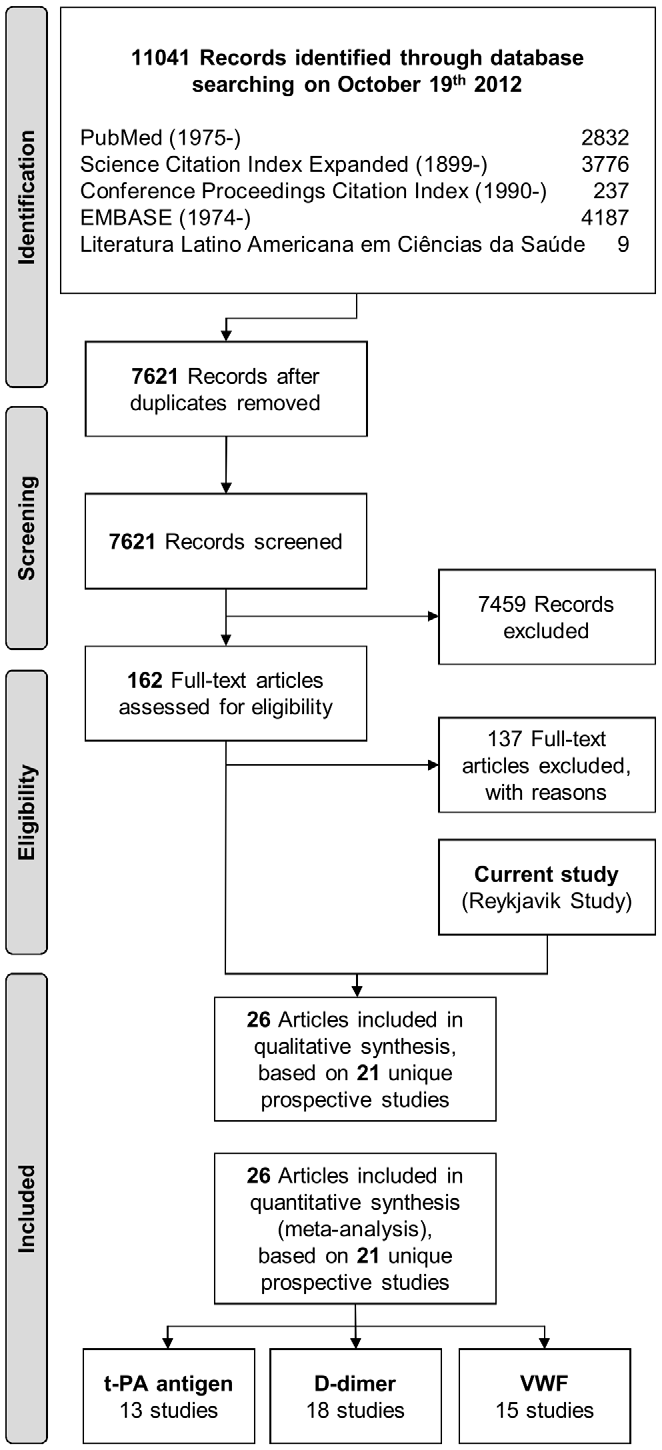
Study flow diagram of the updated meta-analyses. The figure was designed based on the 2009 PRISMA flow diagram template (available from http://www.prisma-statement.org/statement.htm).

All analyses were restricted to participants with complete information on t-PA antigen, D-dimer and VWF. Normal distributions were achieved by taking natural logarithms (log_e_) of positively skewed variables. Odds ratios (ORs) per 1 standard deviation (SD) higher log_e_ t-PA antigen, log_e_ D-dimer and log_e_ VWF were calculated using unconditional logistic regression. The SDs for baseline levels of log_e_ t-PA antigen, log_e_ D-dimer and log_e_ VWF were 0.5, 1.0 and 0.4, corresponding to 1.6-, 2.7- and 1.5-fold differences on the original scale (ie, *e* ^0.5^, *e* ^1.0^ and *e* ^0.4^). To assess the independence of associations from conventional cardiovascular risk factors, ORs were adjusted progressively for age, sex, period of recruitment, smoking, body mass index, history of diabetes, systolic blood pressure, total cholesterol and log_e_ triglycerides. To assess shapes of associations, adjusted ORs were calculated across fifths of the baseline levels of t-PA antigen, D-dimer and VWF in controls and 95% confidence intervals (CIs) were estimated from the floated variances [15]. Subgroup analyses were pre-specified and investigated by formal tests of interaction. Linear regression was used to assess the association between t-PA antigen, D-dimer and VWF and baseline characteristics among controls. To minimize biases resulting from measurement error, we corrected for regression dilution in supplementary analyses. Regression dilution ratios (RDR), as a measure of long-term within-person variability, were calculated for each characteristic by regressing its serial measurements on baseline levels, adjusted for potential confounding factors [16]. Based on these regression models, predictions of long-term average levels (“usual levels”) of t-PA antigen, D-dimer and VWF for each participant were then used to estimate ORs corrected for within-person variability, as described previously [16]. An updated meta-analysis was conducted of prospective studies, published before October 19^th^ 2012, with more than one year of follow-up in essentially general populations (ie, in cohorts not selected on the basis of pre-existing disease) (**Figure 1** **, Methods S1, Figure S2** and **Table S2**). To enable comparability between study reports, risk estimates and 95% CIs were transformed to a common scale (ie, per 1 SD higher levels of the population’s baseline distribution of the respective marker) using methods previously described [11]. Study-specific risk estimates were pooled by random-effects meta-analyses. Odds and hazard ratios were assumed to approximate the same underlying measure of relative risk (RR). Consistency of findings across studies was assessed with standard *χ* ^2^ tests and the *I* ^2^ statistic [17]. Subgroup analyses were conducted using meta-regression across pre-specified study-level characteristics [18]. Evidence of publication bias was assessed with funnel plots, Egger’s test [19], and by comparing pooled results from studies involving at least 500 CHD cases with pooled results from smaller studies. All analyses were performed using Stata release 12.1 (StataCorp, College Station, Texas). Statistical tests were two-sided and used a significance level of *P*<0.05.

## Results

### Baseline Characteristics and Cross-sectional Associations in the Reykjavik Study

In addition to significant differences between cases and controls with respect to conventional risk factors in the Reykjavik Study, baseline concentrations of t-PA antigen and VWF were significantly higher in cases than in controls, while there were no significant differences in D-dimer concentration (**Table 1**). Baseline levels of t-PA antigen, D-dimer and VWF were significantly correlated with each other, with t-PA antigen and D-dimer being inversely associated (**Table 1** and **Figures S3** to **S5**). All three markers were positively associated with age, smoking and inflammatory markers. Male sex and baseline measurements of body mass index, blood pressure, total cholesterol, log_e_ triglycerides, hematocrit, hemoglobin and log_e_ erythrocyte sedimentation rate showed positive correlations with levels of t-PA antigen, but negative correlations with D-dimer. In comparison, age- and sex-adjusted cross-sectional correlations of VWF were generally weaker.

**Table 1:** Baseline characteristics of coronary heart disease cases and matched controls in the Reykjavik Study, and correlations with t-PA antigen, D-dimer and VWF.

Variable	Summary of baseline values					Cross-sectional correlation with hemostatic markers		
	Cases		Controls		P value	Adjusted percentage difference (95% CI)†		
	n	Mean (SD), median (IQR),or *n* (%)	n	Mean (SD), median (IQR),or *n* (%)		t-PA antigen	D-dimer	VWF
**Questionnaire-based**								
Age, years	1925	54.2 (8.7)	3616	55.2 (9.0)	Matched	8% (6 to 11)	24% (19 to 29)	9% (7 to 11)
Male sex	1925	1347 (70%)	3616	2441 (68%)	Matched	23% (19 to 27)	−28% (−33 to −23)	3% (1 to 6)
Current smoker	1925	1153 (60%)	3616	1751 (48%)	<0.0001	4% (1 to 7)	7% (0 to 14)	5% (3 to 8)
History of diabetes	1925	51 (3%)	3616	58 (2%)	0.011	−3% (−15 to 10)	−10% (−30 to 15)	6% (−5 to 17)
**Physical measurements**								
Body mass index, kg/m^2^	1925	26.0 (3.9)	3616	25.4 (3.7)	<0.0001	20% (18 to 21)	−16% (−18 to −13)	1% (0 to 2)
Systolic blood pressure, mmHg	1925	146.7 (21.7)	3616	141.9 (20.1)	<0.0001	11% (9 to 13)	−6% (−9 to −3)	1% (0 to 3)
Diastolic blood pressure, mmHg	1924	90.2 (11.0)	3615	87.5 (10.8)	<0.0001	13% (12 to 15)	−8% (−11 to −5)	1% (−1 to 2)
**Lipid markers**								
Total cholesterol, mmol/L	1925	6.9 (1.2)	3616	6.4 (1.1)	<0.0001	7% (5 to 9)	−5% (−8 to −2)	0% (−1 to 1)
Triglycerides, mmol/L*	1925	1.1 (0.9−1.5)	3616	1.0 (0.8−1.4)	<0.0001	21% (19 to 23)	−14% (−17 to −11)	2% (0 to 3)
Lipoprotein(a), mg/L*	1917	115 (39−283)	3612	82 (25−199)	<0.0001	−3% (−5 to −2)	1% (−2 to 4)	0% (−1 to 1)
**Inflammatory markers**								
Interleukin 6, ng/L*	1658	2.1 (1.4–3.4)	3060	1.9 (1.2–2.9)	<0.0001	5% (4 to 7)	13% (9 to 17)	5% (4 to 6)
C-reactive protein, mg/L*	1906	1.7 (0.8–3.5)	3573	1.2 (0.6–2.7)	<0.0001	13% (11 to 14)	6% (3 to 9)	8% (7 to 9)
**Rheological markers**								
Hematocrit, %	1925	44.7 (4.7)	3616	44.1 (4.5)	<0.0001	10% (9 to 12)	−8% (−12 to −5)	0% (−2 to 1)
Hemoglobin, g/L	1917	148.3 (13.1)	3599	145.9 (13.1)	<0.0001	16% (14 to 18)	−14% (−17 to −11)	1% (−1 to 2)
ESR, mm/hr*	1838	8.0 (4.0–15.0)	3427	7.0 (3.0–13.0.)	<0.0001	3% (1 to 5)	16% (12 to 20)	9% (7 to 10)
**Hemostatic markers**								
t-PA antigen, ng/mL*	1925	13.9 (9.9–18.8)	3616	12.6 (8.8–17.2)	<0.0001	–	−15% (−18 to −12)	6% (5 to 8)
D-dimer, ng/mL*	1925	115 (60–246)	3616	121 (64–241)	0.280	−8% (−10 to −7)	–	7% (6 to 8)
VWF, IU/dL*	1925	107 (81–141)	3616	106 (78–136)	0.008	7% (6 to 9)	17% (14 to 21)	–
**Others**								
Serum creatinine (µmol/L)	1907	77 (20)	3587	75 (13)	<0.001	7% (5 to 9)	0% (−4 to 3)	3% (1 to 5)

Abbreviations: ESR, erythrocyte sedimentation rate; IQR, inter-quartile range.

* Means (SDs) of log_e_ transformed values in cases and controls were 0.2 (0.5) and 0.0 (0.4) for triglycerides; 4.4 (1.6) and 4.0 (1.7) for lipoprotein (a); 0.8 (0.8) and 0.7 (0.8) for interleukin 6; 0.5 (1.1) and 0.2 (1.1) for C-reactive protein; 2.0 (1.0) and 1.9 (1.0) for erythrocyte sedimentation rate; 2.6 (0.5) and 2.5 (0.5) for t-PA antigen; 4.8 (1.0) and 4.8 (1.0) for D-dimer; and 4.7 (0.4) and 4.6 (0.4) for VWF.

† Percentage differences and 95% CIs were calculated per 1 SD higher level or compared to the reference category of variables shown in the left column (adjusted for age, sex and period of recruitment).

### Associations with Incident CHD in the Reykjavik Study

**Figure 2:**
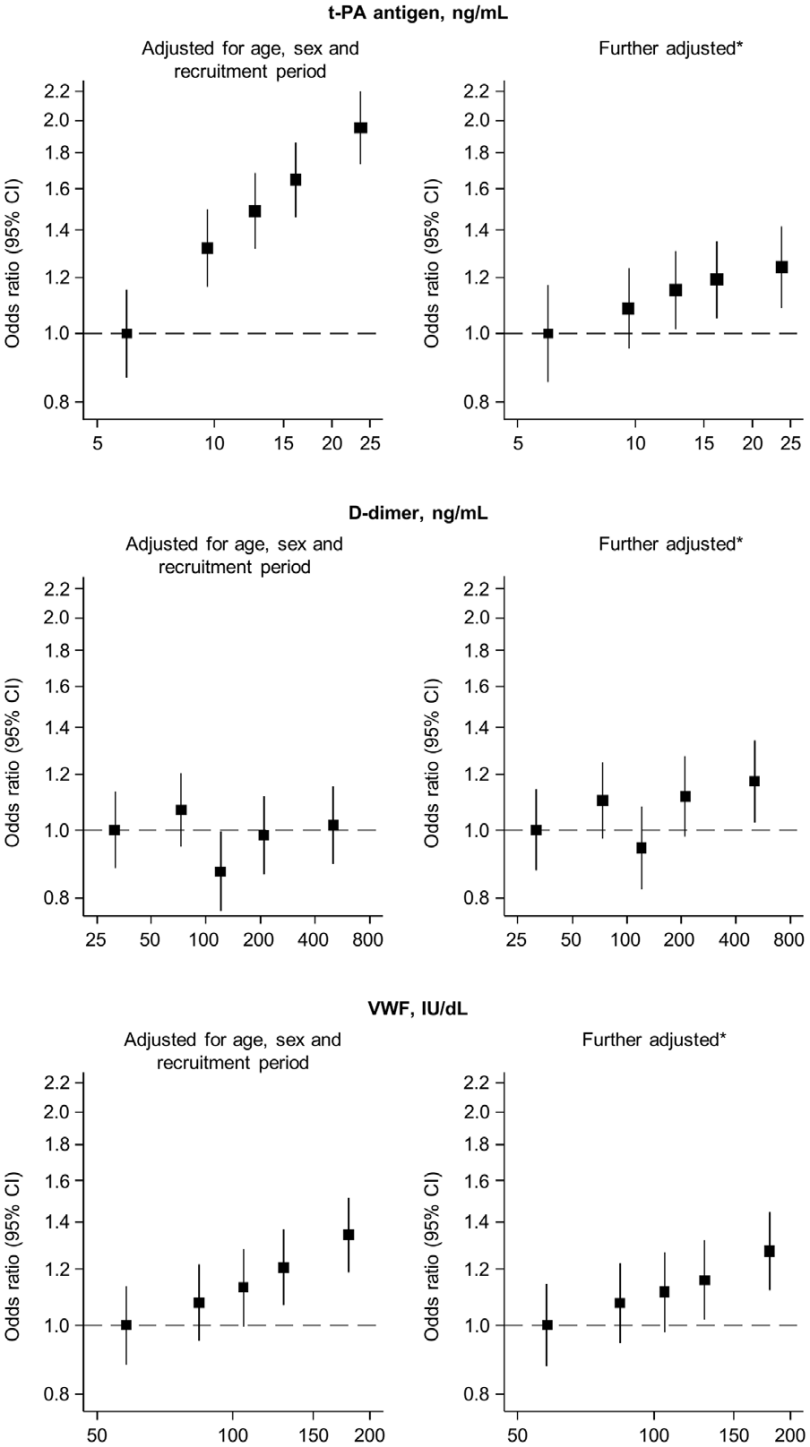
Associations of baseline t-PA antigen, D-dimer and VWF with coronary heart disease risk (Reykjavik Study). Odds ratios (95% CI) for coronary heart disease are shown by fifths of baseline t-PA antigen, D-dimer and VWF, plotted against the geometric mean level in each category on a log-doubling scale. *Adjusted for age, sex, period of recruitment, smoking status, body mass index, systolic blood pressure, history of diabetes, total cholesterol and log_e_ triglycerides.

**Figure 3:**
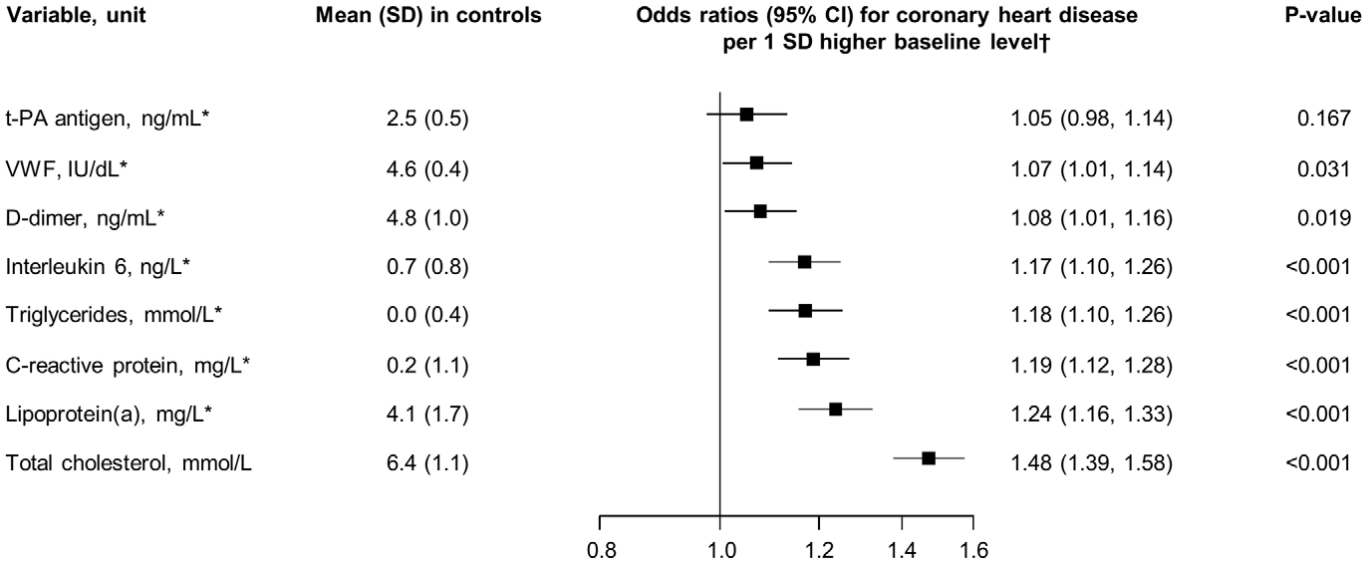
Head-to-head comparison of associations of various baseline variables with coronary heart disease risk (Reykjavik Study). *Values were log_e_ transformed for analysis. †Adjusted for age, sex, period of recruitment, smoking status, body mass index, systolic blood pressure, history of diabetes, total cholesterol and log_e_ triglycerides.

After adjustment for age, sex and period of recruitment, baseline levels of t-PA antigen were apparently log-linearly related to CHD risk (**Figure 2**), yielding an OR of 1.25 (1.18, 1.33) per 1 SD higher level (**Table 2**). This OR fell to 1.07 (0.99, 1.14) after further adjustment for several non-lipid and lipid risk factors. VWF was also log-linearly associated with CHD risk (OR = 1.11 [1.05, 1.18] minimally adjusted; 1.08 [1.02, 1.15] further adjusted). However, there was no evidence of a significant association between baseline levels of D-dimer and CHD risk (OR = 1.01 [0.95, 1.07] minimally adjusted; 1.06 [1.00, 1.13] further adjusted). In a head-to-head comparison, t-PA antigen, D-dimer and VWF were more weakly associated with incident CHD than several established and emerging markers (**Figure 3**). ORs did not vary substantially when further adjustment was made for C-reactive protein (**Table 2**). Apart from a possibly weaker association of t-PA antigen in current smokers compared with non-smokers (P = 0.003), ORs for CHD did not vary in clinically relevant subgroups defined by diabetes status, lipids, obesity, or other individual-level characteristics (**Figure S6**).

**Table 2:** Association of baseline levels of t-PA antigen, D-dimer and VWF with coronary heart disease in the Reykjavik Study (1925 cases, 3616 controls).

Adjustment	Odds ratios for coronary heart disease (95% CI) per 1 SD higher level		
	t-PA antigen	D-dimer	VWF
Adjusted for age, sex and period of recruitment	1.25 (1.18, 1.33)	1.01 (0.95, 1.07)	1.11 (1.05, 1.18)
+ non-lipid risk factors*	1.14 (1.07, 1.22)	1.04 (0.98, 1.10)	1.08 (1.02, 1.15)
+ lipid risk factors†	1.07 (0.99, 1.14)	1.06 (1.00, 1.13)	1.08 (1.02, 1.15)

* Smoking status, body mass index, systolic blood pressure and any history of diabetes mellitus at baseline.

† Total cholesterol and log_e_ triglycerides.

When analyses were restricted to participants with complete information on C-reactive protein (1906 cases, 3573 controls), the odds ratios (95% CI) per 1 SD higher value of t-PA antigen were 1.26 (1.18, 1.33) when adjusting for age, sex and period of recruitment, 1.15 (1.07, 1.22) when additionally adjusting for non-lipid risk factors, 1.07 (0.99, 1.14) when additionally adjusting for lipid risk factors and 1.04 (0.97, 1.11) when additionally adjusting for C-reactive protein. Corresponding odds ratios were 1.01 (0.95, 1.07), 1.04 (0.98, 1.11), 1.07 (1.00, 1.14) and 1.05 (0.99, 1.12) for D-dimer and 1.12 (1.05, 1.18), 1.09 (1.02, 1.15), 1.09 (1.03, 1.16) and 1.06 (1.00, 1.13) for VWF.

### Correction for Long-term within-person Variability

In up to 371 participants who provided paired measurements at baseline and a mean (SD) of 11.6 (1.3) years later, age- and sex-adjusted RDRs were 0.47 (95% CI 0.38, 0.56) for t-PA antigen, 0.30 (0.22, 0.38) for D-dimer and 0.55 (0.47, 0.63) for VWF (**Table S3**). The within-person variability over 11.6 years in the current study was somewhat greater than reported in previous studies with measurements taken 5 or fewer years apart (p≤0.007 for trend with time, **Figure S7**). After correction for within-person variability in levels of the exposures, the ORs per 1 SD higher level were 1.15 (1.01, 1.32) for t-PA antigen, 1.11 (0.99, 1.25) for D-dimer 1.10 (1.03, 1.19) for VWF.

### Systematic Review and Updated Meta-analyses

**Figure 4:**
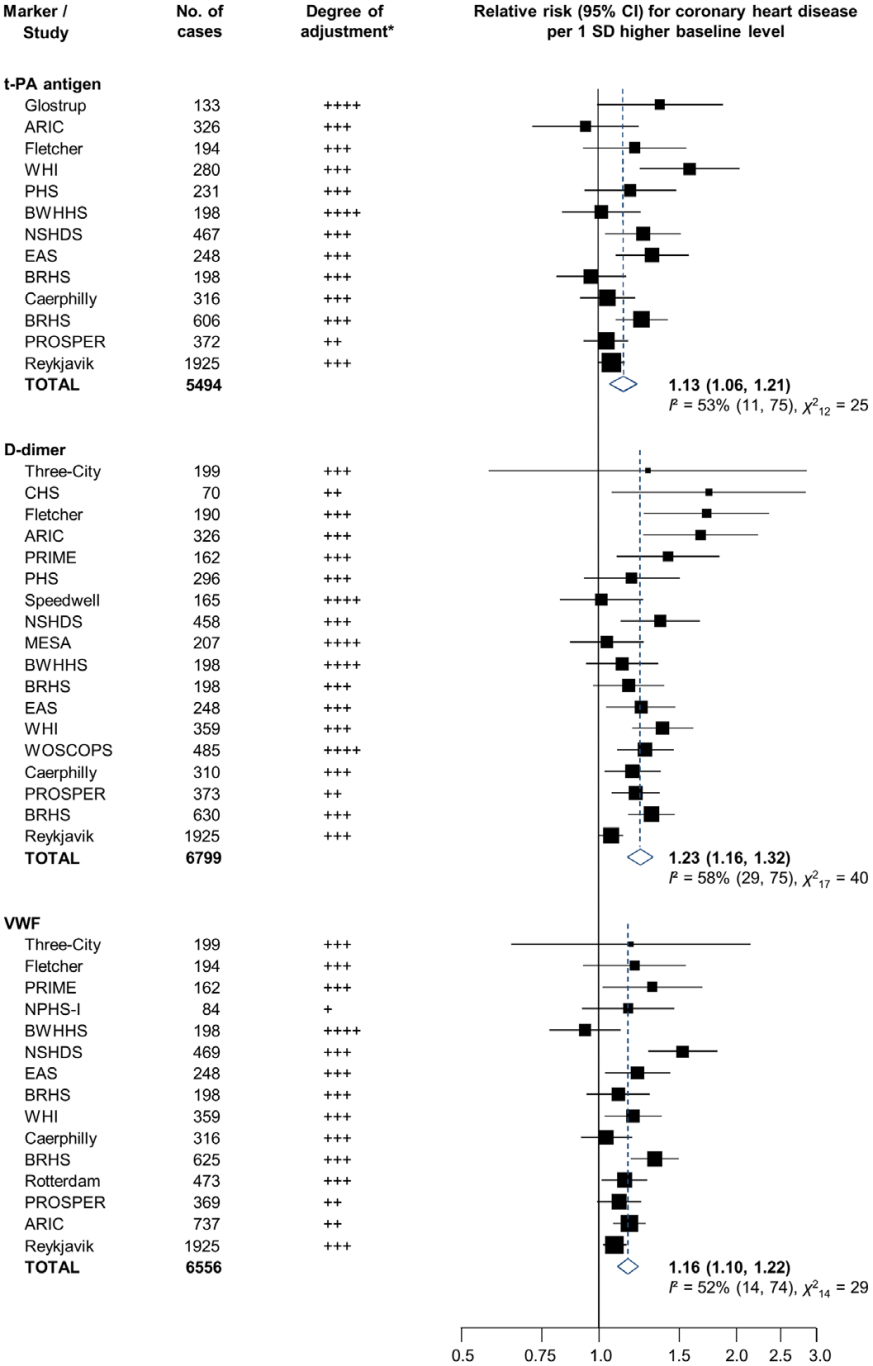
Meta-analyses of reported associations of t-PA antigen, D-dimer and VWF with coronary heart disease risk in prospective population-based studies. Study acronyms are explained in the legend of **Table 3**. Summary estimates were calculated using random effects models. *Degree of adjustment:+minimally adjusted (typically adjusted for age and sex only);++plus adjustment for at least one non-lipid marker;+++plus adjustment for at least one lipid marker;++++plus adjustment for at least one inflammatory marker. Where studies reported relative risks with more than one level of statistical adjustment, the most adjusted estimate was used (least adjusted estimates are reported in **Figure S8**).

Together with the current study, 21 relevant published prospective studies were identified (13 with t-PA antigen, 18 with D-dimer and 15 with VWF measurement) [7]–[10], [20]–[40]. Study characteristics and references are provided in **Table 3**. All but one were based in the United States or Europe. Most identified participants in population registers or in occupational settings and reported on incident MI or CHD death outcomes (mean follow-up ranged from about 2.4 to 19.4 years). Measurements generally involved enzymatic immunoassays, performed in plasma (17 studies) or serum (4 studies), thawed after long-term storage at −20°C (4 studies), −40°C (1 study), −70 or −80°C (14 studies) or in liquid nitrogen (2 studies). Reported levels of t-PA antigen and VWF were consistent across studies, while levels of D-dimer varied substantially (ranging from around 10 to over 600 IU/dL). A combined analysis of available data yielded adjusted RRs of 1.13 (1.06, 1.21), 1.23 (1.16, 1.32) and 1.16 (1.10, 1.22) per 1 SD higher baseline levels of t-PA antigen (5494 CHD cases), D-dimer (6799 CHD cases) and VWF (6556 CHD cases), respectively (**Figure 4**). There was some evidence of heterogeneity in RRs, but little of it was explained by recorded study-level characteristics (**Figure S9**). There was some evidence that smaller studies of D-dimer reported more striking findings (Egger’s test, *P* = 0.004; **Figure S10**), but this trend was not apparent when results from studies with 500 CHD cases or more were compared with those from smaller studies in the pre-specified subgroup analyses (**Figure S9**).

**Table 3:** Characteristics of 21 published prospective studies of t-PA antigen, D-dimer and VWF.

								Blood sample					Assay manufacturer			Mean level		
Study [Reference]	Location	Population source	Year of baseline	Age range at baseline	Male, %	Mean follow-up, years	Endpoint definition	Fasted	Specimen type	Storage temp	No. of cases	No. of controls	t-PA antigen	D-Dimer	VWF	t-PA antigen, ng/mL	D-Dimer, ng/mL	VWF, IU/dL or %
**Current study**																		
Reykjavik	Iceland	Population register	1969–96	33–86	70	19.4†	CHD	Yes	Serum	−20	1925	3616	Biopool	Hyphen	Dako	13.7	198	112
**Previously published**																		
ARIC [20]	USA	Household listings	1987–89	45–64	43	4.3	CHD, CS	Yes	Plasma	−70	326	692	Diagnostica	Diagnostica		7.6||	303||	
ARIC [21]	USA	Household listings	1987–89	45–64	43	12.4	Nonfatal MI	Yes	Plasma	−70	737	13272			AmB			NR
BRHS* [9]	UK	GP lists	1978–80	40–59	100	18‡	CHD	No	Serum	−20	606	1227	Biopool			10.4||		
BRHS* [7]	UK	GP lists	1978–80	40–59	100	18‡	CHD	No	Serum	−20	630	1269		AGEN			130	
BRHS* [8]	UK	GP lists	1978–80	40–59	100	18‡	CHD	No	Serum	−20	625	1266			Dako			113
BRHS* [10]	UK	GP lists	1998–00	60–79	100	7	CHD	Yes	Plasma	−70	198	2809	Biopool	Biopool	Dako	10.8	80	137
BWHHS [22]	UK	Population register	1999–01	60–79	0	4.7†	CHD, CS, AP	Yes	Plasma	−80	198	3384	Biopool	Biopool	Dako	7.9||	88||	139||
Caerphilly [23]	UK	Electoral rolls	1984–88	49–65	100	13.4†	CHD	Yes	Plasma	−70	316	1665	Biopool		Dako	10.9||		113||
Caerphilly [23]	UK	Electoral rolls	1984–88	49–65	100	13.4†	CHD	Yes	Plasma	−70	310	1621		AGEN			12||	
CHS [24]	USA	Medicare lists	1989–90	65+	61	2.4	CHD	Yes	Plasma	−70	70	70		In-house			NR	
EAS [25]	UK	GP lists	1988	55–74	53	17	CHD	Yes	Plasma	−40	248	1177	Biopool	AGEN	Dako	7.0†	80†	105†
Fletcher [26]	NZ	Occupational/electoral	1992–94	19–86	72	5.5†	CHD	No	Plasma	−70	194	399	Hyphen		Dako	4.7		127
Fletcher [26]	NZ	Occupational/electoral	1992–94	19–86	72	5.5†	CHD	No	Plasma	−70	190	387		R&D Sys			253	
Glostrup [27]	Denmark	Population register	1976–84	30–60	73	7–15§	CHD	Yes	Serum	−20	133	258	Biopool			7.8		
MESA [28]	USA	General population	2000–02	45–84	47	4.6†	CHD	Yes	Plasma	−70	207	6184		DStago			205†	
NPHS-I [29]	UK	Occupational register	1978–84	40–64	100	10.1	CHD	Yes	Serum	−196	84	933			In-house			77%
NSHDS [30]	Sweden	General population	1985–99	25–74	79	14	CHD	Yes	Plasma	−80	467	893	Biopool			8.0		
NSHDS [30]	Sweden	General population	1985–99	25–74	79	14	CHD	Yes	Plasma	−80	458	882		Hyphen			174	
NSHDS [30]	Sweden	General population	1985–99	25–74	79	14	CHD	Yes	Plasma	−80	469	895			Dako			141
PHS [31]	USA	Occupational register	1982–84	40–84	100	5	CHD	No	Plasma	−80	231	231	Biopool			9.2		
PHS [32]	USA	Occupational register	1982–84	40–84	100	5	CHD	No	Plasma	−80	296	296		Biopool			49	
PRIME [33]	France/NI	General population	1991–93	50−59	100	5‡	CHD	Yes	Plasma	–196	162	324		DStago	DStago		255†	114†
PROSPER [34]	SCO/IRE/NL	Primary care screening	1998	70–82	48	3.2	CHD	Yes	Plasma	−80	372	2750	Hyphen			11.0		
PROSPER [34]	SCO/IRE/NL	Primary care screening	1998	70–82	48	3.2	CHD	Yes	Plasma	−80	373	2806		Hyphen			270	
PROSPER [34]	SCO/IRE/NL	Primary care screening	1998	70–82	48	3.2	CHD	Yes	Plasma	−80	369	2739			Dako			139
Rotterdam [35]	NL	Population register	1989–93	55+	39.8	6.4	CHD, CS	Yes	Plasma	−80	473	5328			Dako			130
Speedwell [36]	UK	GP list	1982–85	49–67	100	6.25	CHD	No	Plasma	−20	165	1554		Biopool			41||	
Three-City [37]	France	Electoral rolls	1999–01	65+	39.5	4‡	CHD, CS, AP	Yes	Plasma	−80	199	1053		DStago	DStago		576||	130%||
WHI [38]	USA	Population register	1993–98	50–79	0	2.9	CHD	Yes	Plasma	−70	280	280	AmD			7.4†		
WHI [39]	USA	Population register	1993–98	50–79	0	4‡	CHD	Yes	Plasma	−70	359	820		AmD	AmD		300†	93%†
WOSCOPS [40]	UK	Heart screening clinic	1989–91	45–64	100	6‡	CHD, CS	Yes	Plasma	−70	485	934		Biopool			60	

**Abbreviations:** **AmB**, American Bioproducts; **AmD**, American Diagnostica; **AP**, angina pectoris; **ARIC**, Atherosclerosis Risk in Communities Study; **BRHS**, British Regional Heart Study; **BWHHS**, British Women’s Heart and Health Study; **Caerphilly**, Caerphilly Prospective Study; **CHD**, coronary heart disease endpoint (composed of nonfatal myocardial infarction and coronary death); **CHS**, Cardiovascular Health Study; **CS**, coronary surgery; **DStago**, Diagnostica Stago; **EAS**, Edinburgh Artery Study; **ELISA**, enzyme-linked immunosorbent assay; **FHS**, Framingham Heart Study; **Fletcher**, Fletcher Challenge Study; **Glostrup**, Glostrup population studies; **IRE**, Ireland; **IT**, immunoturbidometry; **MESA**, Multi-Ethnic Study of Atherosclerosis; **MI**, myocardial infarction; **NPHS-I**, Northwick Park Heart Study I; **NSHDS**, Northern Sweden Health and Diseases Study cohort; **PHS**, Physicians’ Health Study; **PRIME**, Prospective Epidemiological Study of Myocardial Infarction; **PROSPER**, Prospective Study of Pravastatin in the Elderly at Risk; **NI**, Northern Ireland; **NL**, Netherlands; **NR**, not reported; **NZ**, New Zealand; **R&D Sys**, R&S Systems; **RE**, rocket electrophoresis; **Rotterdam**, Rotterdam Study; **SCO**, Scotland; **Speedwell**, Speedwell Study; **Three-City**, Three-City cohort study; **WHI**, Women’s Health Initiative; **WOSCOPS**, The West of Scotland Coronary Prevention Study.

* Reports with two different study baselines and non-overlapping follow-up periods are available.

† Median.

‡ Maximum.

§ Range.

|| Geometric mean.

## Discussion

In the Reykjavik Study, the largest population-based study of t-PA antigen, D-dimer and VWF to date, we observed at most weak associations of these markers with the risk of CHD, when adjusting for a panel of conventional cardiovascular risk factors. However, in the context of the updated meta-analyses that involved up to ten times as much data as previously reported [7]–[9], higher levels of t-PA antigen, D-dimer and VWF at baseline were compatible with modest increases in risk. As suggested by previous reports, while t-PA antigen was positive associated with various risk factors, including male sex, blood pressure, body mass index, triglycerides, inflammatory markers and rheological markers [9], [20], D-dimer was inversely associated with these risk factors and also inversely associated with t-PA antigen levels [7]. This inverse association could arise because t-PA antigen levels largely reflect circulating t-PA/PAI-1 complexes, and higher PAI-1 levels reduce endogenous fibrinolysis and hence D-dimer levels. Finally, baseline VWF levels were largely independent from other cardiovascular risk factors (with weak correlations with C-reactive protein and smoking status).

Although the combined estimates from the current study and previous reports were compatible with modest increases in risk, the relationships of these three markers with CHD risk remain uncertain, as evidenced by: the comparatively wide confidence intervals around the pooled RRs; a tendency for more extreme associations in smaller studies suggesting the likelihood of some exaggeration in the overall estimates; and most importantly, potential residual confounding, as the meta-analyses pooled associations adjusted for baseline levels of confounders only [41]. The associations of t-PA antigen, D-dimer and VWF with first-ever MI or CHD death may therefore be more modest than previously reported, but the current findings do not, of course, preclude stronger associations in high-risk populations [42], [43] or with different vascular outcomes [44], [45].

To help clarify whether activated coagulation and fibrinolysis play an etiological role in the development of CHD, future investigations should involve complementary approaches to help judge causality, such as study of potential interrelations with ABO(H) blood groups [46], [47]; investigation of more direct markers such as t-PA activity or VWF multimer patterns [6]; and mendelian randomization studies using genetic variants as potentially unconfounded proxies for circulating levels of these markers [48]–[51]. In particular, VWF levels may be a plausible mediator of the association of non-O blood groups with CHD and other thrombotic disorders [52].

Furthermore, the within-person variability of these hemostatic markers is greater than previously reported and highlights the importance of correction for variability in long-term prospective studies and the potential advantages of identifying genetic variants as proxies for these error-prone biomarkers [53]. While literature-based data indicate a declining self-correlation over time, suggesting that single “snapshot” baseline measurements do not perfectly capture long-term “usual” levels, the magnitude of this remains to be determined in detail. Information on the repeatability of such measurements over time is currently available on only around 2000 individuals in aggregate and is based predominantly on summary findings from published reports and not individual data.

The strengths and limitations of the current report merit careful consideration. Our new data from the Reykjavik Study involve more than three times as many cases of first-ever CHD as in the previous largest reports [7]–[9]. Participants were identified in population registers, had high response and follow-up rates, used robust methods to ascertain CHD outcomes and minimized potential biases by exclusion of participants with prevalent cardiovascular disease. Concomitant measurements of several factors enabled direct comparisons with different markers and allowed adjustment for a range of possible confounders, although we acknowledge that data on some biological factors was lacking [54], [55]. Repeat measurements several years apart enabled assessment of and allowance for long-term within-person variability, but no data were available to account for short-term variability, such as diurnal or seasonal fluctuations [53], [56]. Although the current study involved blood storage at −20°C for a median time of 29 years (range 4 to 33 years), the distributions of t-PA antigen, D-dimer and VWF were broadly in keeping with those reported previously, as exemplified by the WHO-MONICA optional Haemostasis Study of 11 centers across Europe that also included Iceland [57]. Furthermore, the generally similar correlations at baseline with cardiovascular risk factors in this and other studies, plus the lack of any significant heterogeneity in associations with CHD risk across studies with different lengths of follow-up or sample storage conditions in the updated meta-analyses, argues against sample degradation as an explanation for our findings. Finally, although the updated meta-analyses included in our report represent the most comprehensive synthesis of the available data to date, they rely on published reports only. Access to individual participant data would permit more consistent comparisons across studies (such as in the outcomes, covariates and subgroups considered).

### Conclusions

Concentrations of t-PA antigen, D-dimer and VWF may be more modestly associated with first-ever CHD events than previously reported. More detailed analysis is required to clarify whether these markers are causal risk factors or simply correlates of coronary heart disease.

## Supporting Information

Figure S1 **Flow-chart of the Reykjavik Study.**
(PDF)

Figure S2 **Search strategy used in the updated meta-analyses.**
(PDF)

Figure S3 **Cross-sectional correlates of baseline levels of t-PA antigen.**
(PDF)

Figure S4 **Cross-sectional correlates of baseline levels of D-dimer.**
(PDF)

Figure S5 **Cross-sectional correlates of baseline levels of VWF.**
(PDF)

Figure S6 **Explorative analysis of effect modification of the associations of baseline levels of t-PA antigen, D-dimer and VWF with coronary heart disease risk in the Reykjavik study.** Continuous variables were categorized based on tertile cut-offs in controls. All models are adjusted for age, sex, period of recruitment, smoking status, body mass index, systolic blood pressure, history of diabetes at baseline, total cholesterol, and loge triglycerides. *P values were calculated using likelihood ratio tests comparing models with and without interaction terms.
(PDF)

Figure S7 **Estimated within-person variability of t-PA antigen, D-dimer and VWF by time since baseline measurement in the published literature.** Abbreviations: ARIC, Atherosclerosis Risk in Communities Study; BRHS, British Regional Heart Study; EAS, Edinburgh Artery Study; Fletcher, Fletcher Challenge Study; Reykjavik, Reykjavik Study. Each point represents unadjusted study- and time-specific estimates of within-person variability (eg, reported correlation coefficients or regression dilution ratios) in paired samples taken some time apart. Grey boxes represent unadjusted estimates. The relative sizes of the boxes are proportional to the inverse of the standard errors.
(PDF)

Figure S8 **Meta-analyses of reported associations of t-PA antigen, D-dimer and VWF with coronary heart disease risk in population-based prospective studies. Where studies reported more than one odds ratio, the least adjusted estimate was used.** Study acronyms are explained in the legend of Table 3. Summary estimates were calculated using random effects models. *Degree of adjustment:+minimally adjusted (typically adjusted for age and sex only);++plus adjustment for at least one non-lipid marker;+++plus adjustment for at least one lipid marker;++++plus adjustment for at least one inflammatory marker. Where studies reported relative risks with more than one level of statistical adjustment, the least adjusted estimate was used (most adjusted estimates are reported in Figure 4).
(PDF)

Figure S9 **Investigation of sources of possible heterogeneity in reported associations of t-PA antigen, D-dimer and VWF with coronary heart disease risk, according to various study-level characteristics.** Summary estimates were calculated using random effects models. *P values are from meta-regression for differences in odds ratios across studies in different groups.
(PDF)

Figure S10 **Funnel plots of reported associations of t-PA antigen, D-dimer and VWF with coronary heart disease risk.** The dotted lines show 95% confidence intervals around the overall summary estimate calculated using fixed effect models. Egger’s test for regression asymmetry: t-PA antigen, P = 0.086; D-dimer, P = 0.004; VWF, P = 0.290.
(PDF)

Table S1 **Comparison of baseline characteristics of coronary heart disease cases, randomly selected controls and the overall Reykjavik cohort.** *Controls were matched to coronary heart disease cases by sex and age (in five-year age bands) and therefore differ from the remainder of the Reykjavik cohort.
(PDF)

Table S2 **PRISMA 2009 Checklist.** From: Moher D, Liberati A, Tetzlaff J, Altman DG, The PRISMA Group (2009). Preferred Reporting Items for Systematic Reviews and Meta-Analyses: The PRISMA Statement. PLoS Med 6(6): e1000097. doi:10.1371/journal.pmed1000097. For more information, visit: www.prisma-statement.org.
(PDF)

Table S3 **Within-person variability of t-PA antigen, D-dimer and VWF plus other measured markers (mean time between repeated measurements, 11.6 years).**
(PDF)

Methods S1 **Systematic review and updated meta-analysis.**
(PDF)
