# Supplementary figures and images for: Hemostatic Factors and Risk of Coronary Heart Disease in General Populations: New Prospective Study and Updated Meta-Analyses
Source: PLoS One. 2013 Feb 7;8(2):e55175. doi: 10.1371/journal.pone.0055175 (PMC3567058; doi:10.1371/journal.pone.0055175)

**Figure S1.** Flow-chart of the Reykjavik Study.

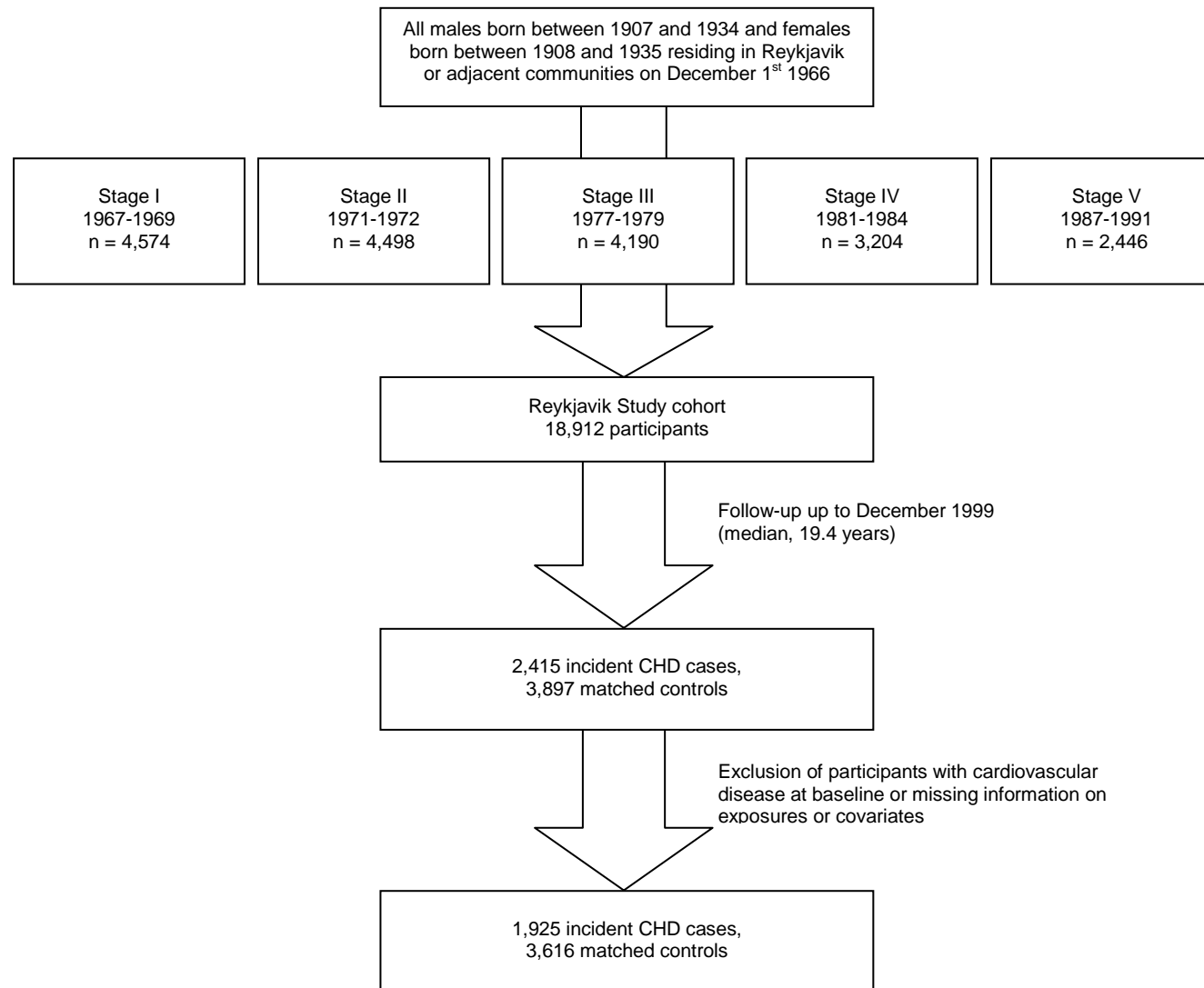

Supplement: Figure S1 — Flow-chart of the Reykjavik Study. (PDF) [file pone.0055175.s001.pdf]

Figure S2. Search strategy used in the updated meta-analyses.

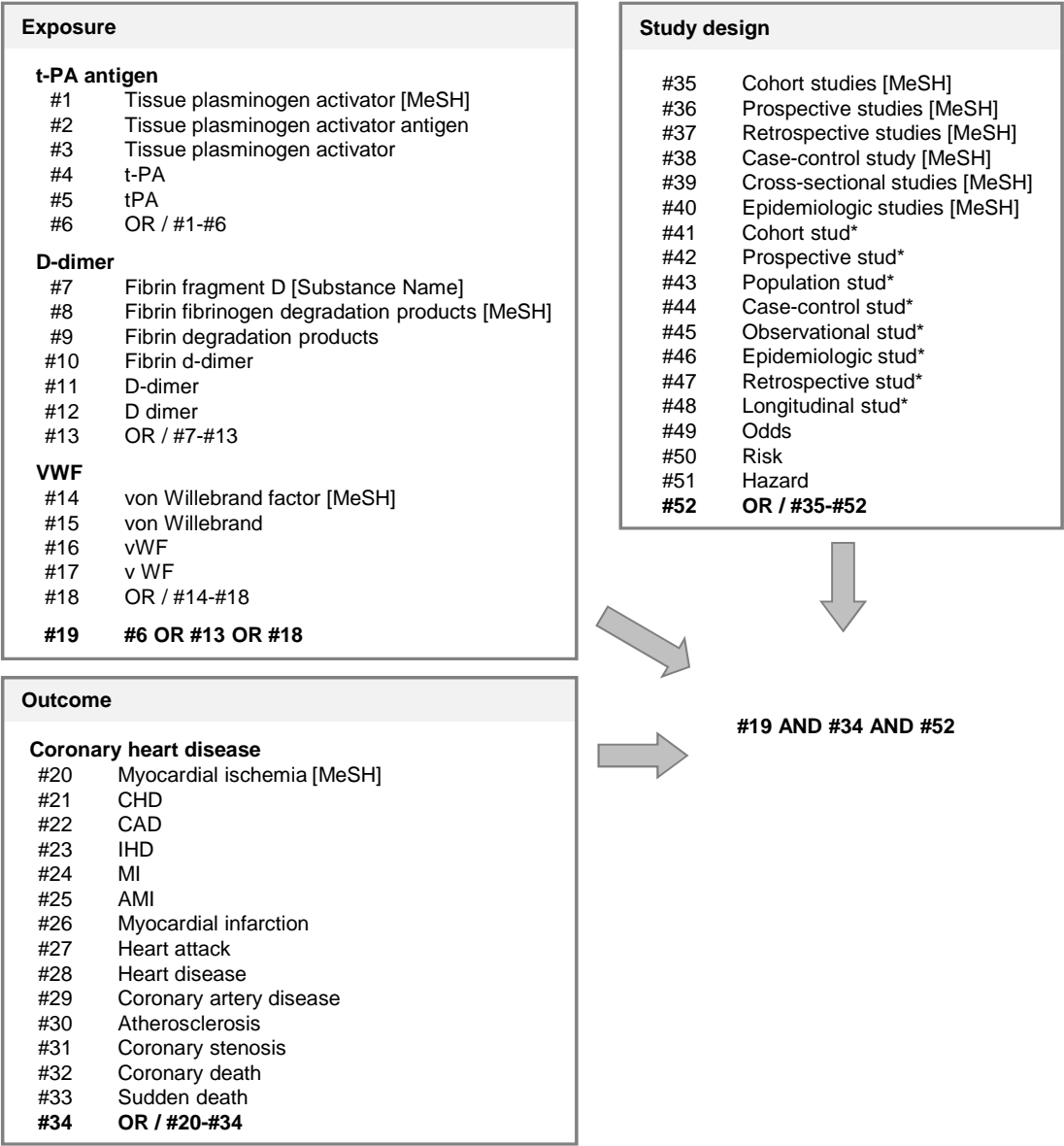

Supplement: Figure S2 — Search strategy used in the updated meta-analyses. (PDF) [file pone.0055175.s002.pdf]
