# Supplementary material for: Hemostatic Factors and Risk of Coronary Heart Disease in General Populations: New Prospective Study and Updated Meta-Analyses
Source: PLoS One. 2013 Feb 7;8(2):e55175. doi: 10.1371/journal.pone.0055175 (PMC3567058; doi:10.1371/journal.pone.0055175)
Supplement: Figure S3 — Cross-sectional correlates of baseline levels of t-PA antigen. (PDF) [file pone.0055175.s003.pdf]

Figure S3. Cross-sectional correlates of baseline levels of t-PA antigen.

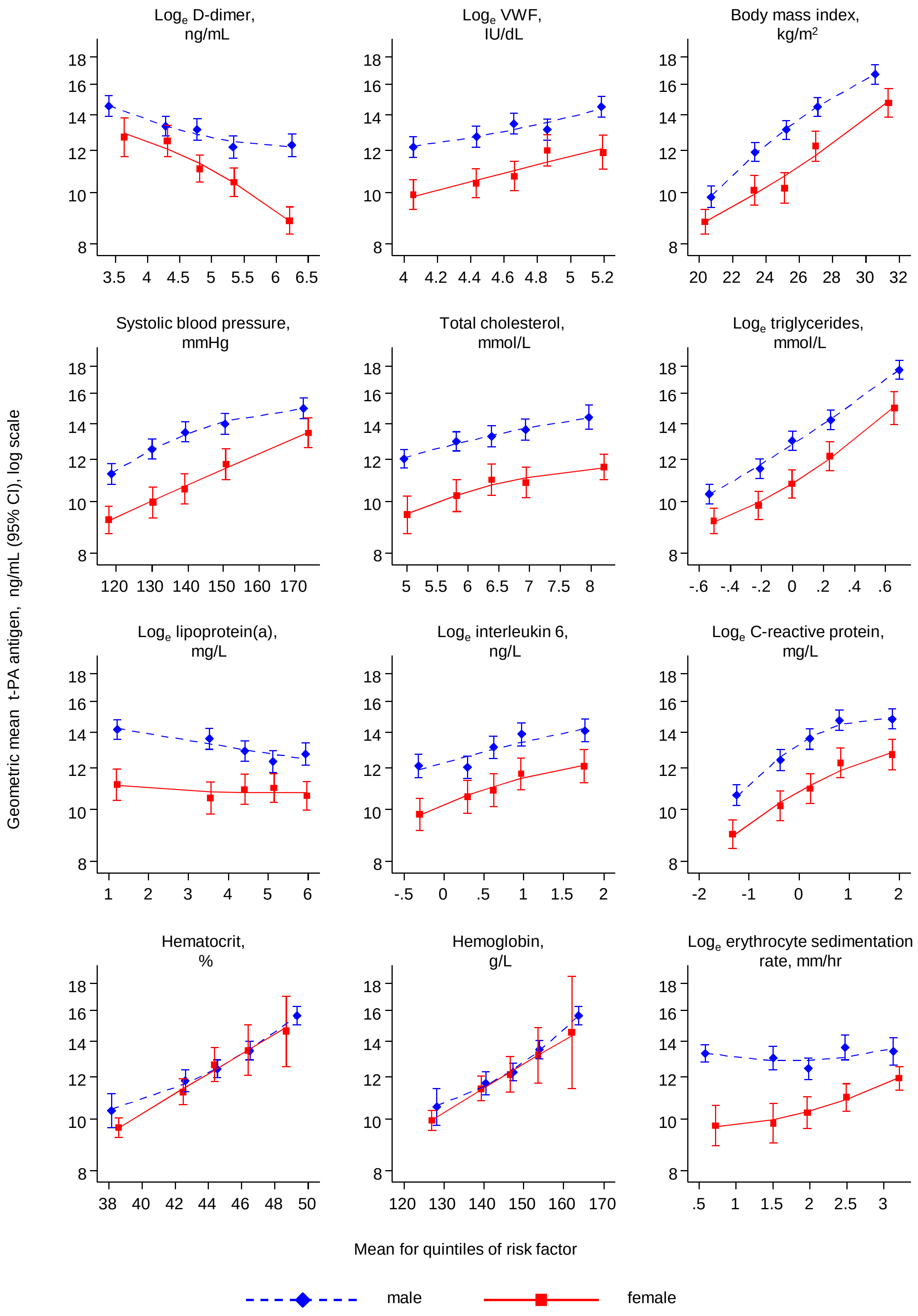

Response means are adjusted to age 55
