# Supplementary material for: Hemostatic Factors and Risk of Coronary Heart Disease in General Populations: New Prospective Study and Updated Meta-Analyses
Source: PLoS One. 2013 Feb 7;8(2):e55175. doi: 10.1371/journal.pone.0055175 (PMC3567058; doi:10.1371/journal.pone.0055175)
Supplement: Figure S4 — Cross-sectional correlates of baseline levels of D-dimer. (PDF) [file pone.0055175.s004.pdf]

Figure S4. Cross-sectional correlates of baseline levels of D-dimer.

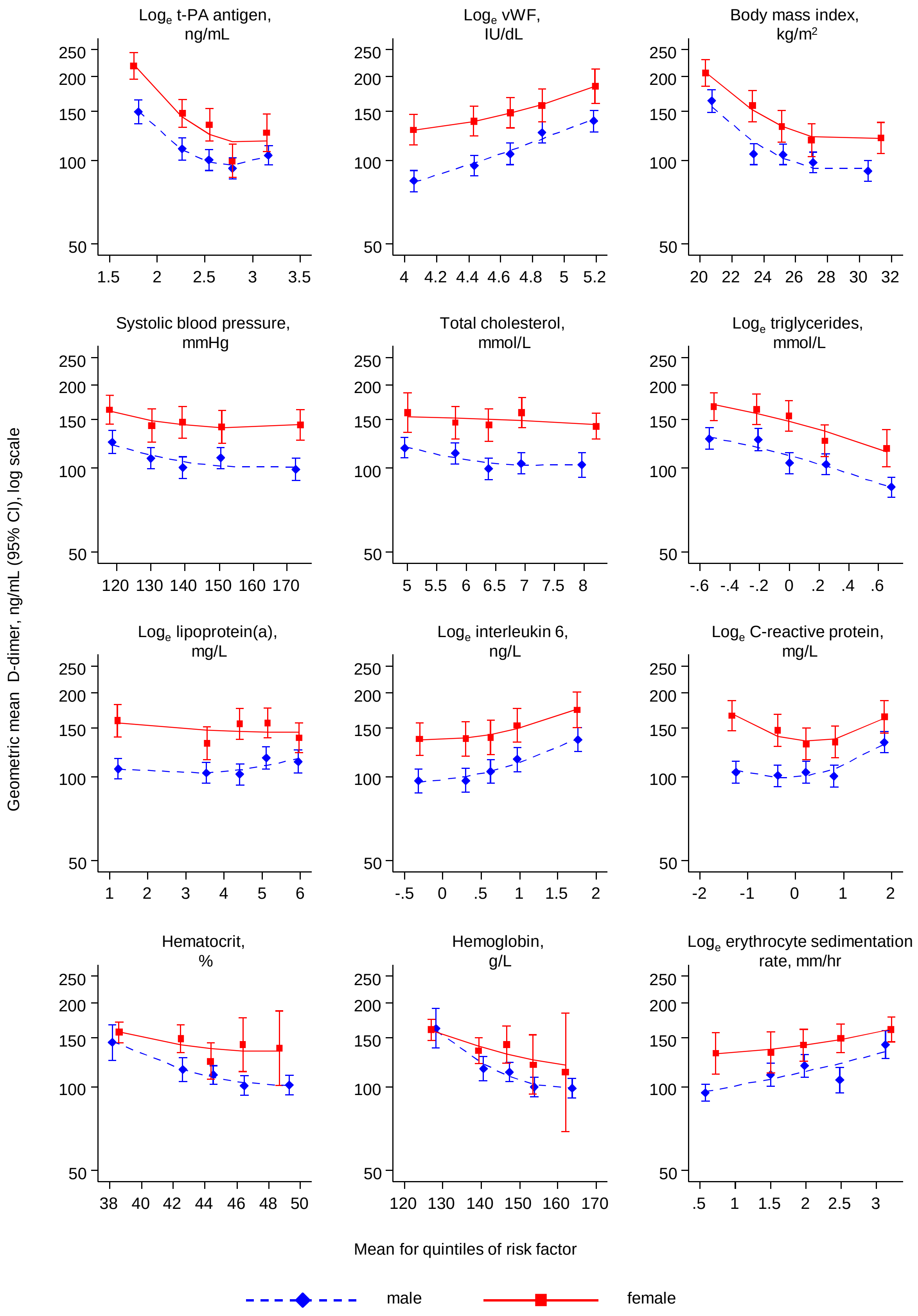

Response means are adjusted to age 55
