# Supplementary material for: Hemostatic Factors and Risk of Coronary Heart Disease in General Populations: New Prospective Study and Updated Meta-Analyses
Source: PLoS One. 2013 Feb 7;8(2):e55175. doi: 10.1371/journal.pone.0055175 (PMC3567058; doi:10.1371/journal.pone.0055175)
Supplement: Figure S6 — Explorative analysis of effect modification of the associations of baseline levels of t-PA antigen, D-dimer and VWF with coronary heart disease risk in the Reykjavik study. Continuous variables were categorized based on tertile cut-offs in controls. All models are adjusted for age, sex, period of recruitment, smoking status, body mass index, systolic blood pressure, history of diabetes at baseline, total cholesterol, and loge triglycerides. *P values were calculated using likelihood ratio tests comparing models with and without interaction terms. (PDF) [file pone.0055175.s006.pdf]

Figure S6. Explorative analysis of effect modification of the associations of baseline levels of t-PA antigen, D-dimer and VWF with coronary heart disease risk in the Reykjavik study.

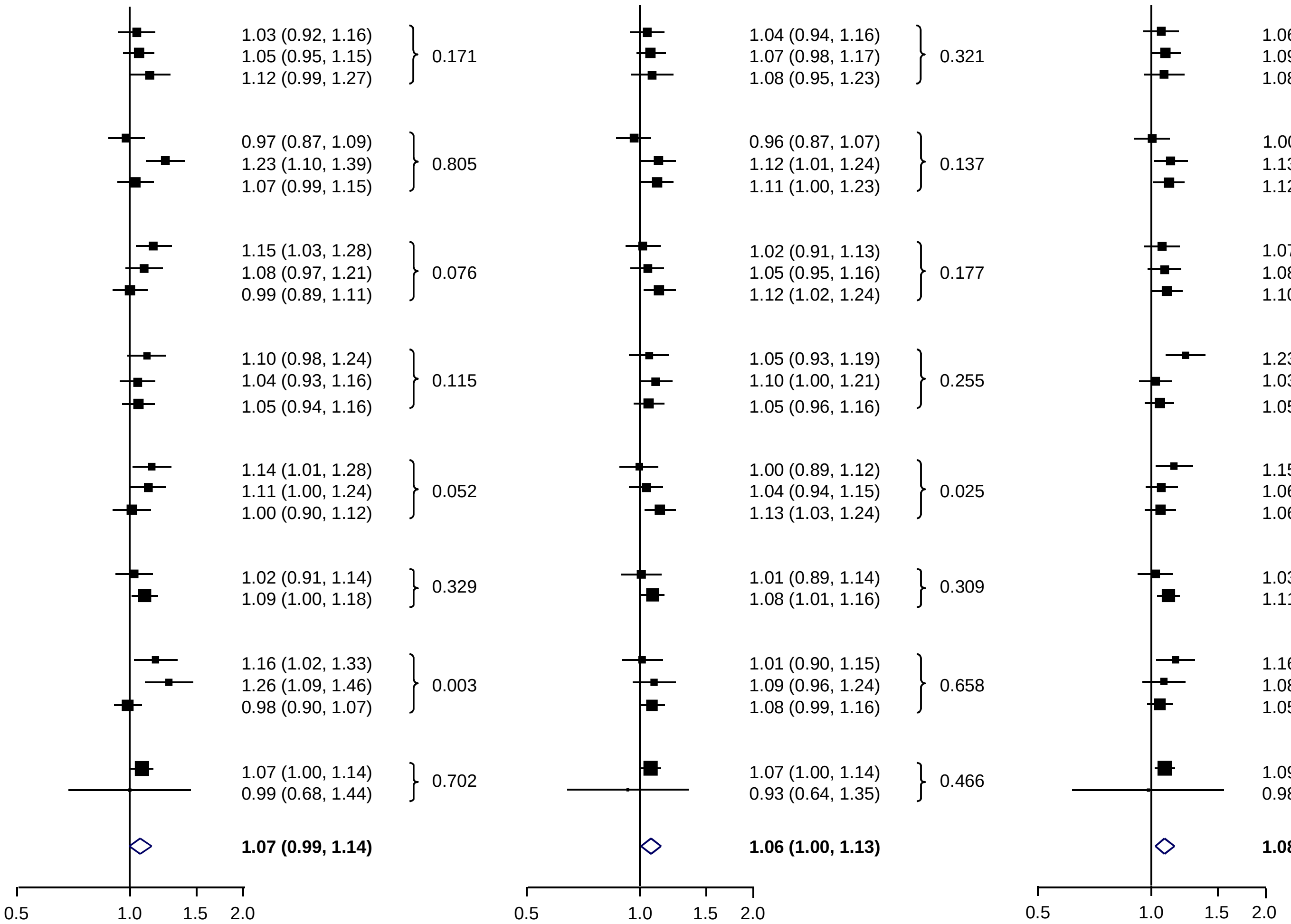

Subgroup	No. of cases	t-PA antigen: Odds ratio for coronary heart disease (95% CI)	t-PA antigen: P value*	D-dimer: Odds ratio for coronary heart disease (95% CI)	D-dimer: P value*	VWF: Odds ratio for coronary heart disease (95% CI)	VWF: P value*
Age							
< 50 years	577	1.03 (0.92, 1.16)	0.171	1.04 (0.94, 1.16)	0.321	1.06 (0.95, 1.18)	0.371
50-59 years	864	1.05 (0.95, 1.15)		1.07 (0.98, 1.17)		1.09 (1.00, 1.19)	
≥ 60 years	484	1.12 (0.99, 1.27)		1.08 (0.95, 1.23)		1.08 (0.96, 1.22)	
Body mass index, kg/m^2							
< 23.7	526	0.97 (0.87, 1.09)	0.805	0.96 (0.87, 1.07)	0.137	1.00 (0.90, 1.12)	0.929
23.7-26.7	643	1.23 (1.10, 1.39)		1.12 (1.01, 1.24)		1.13 (1.02, 1.25)	
≥ 26.7	756	1.07 (0.99, 1.15)		1.11 (1.00, 1.23)		1.12 (1.01, 1.23)	
Systolic blood pressure, mmHg							
< 131.0	506	1.15 (1.03, 1.28)	0.076	1.02 (0.91, 1.13)	0.177	1.07 (0.96, 1.19)	0.537
131.0-148.5	612	1.08 (0.97, 1.21)		1.05 (0.95, 1.16)		1.08 (0.98, 1.20)	
≥ 148.5	807	0.99 (0.89, 1.11)		1.12 (1.02, 1.24)		1.10 (1.00, 1.21)	
Total cholesterol, mmol/L							
< 5.9	365	1.10 (0.98, 1.24)	0.115	1.05 (0.93, 1.19)	0.255	1.23 (1.09, 1.39)	0.140
5.9-6.8	621	1.04 (0.93, 1.16)		1.10 (1.00, 1.21)		1.03 (0.93, 1.14)	
≥ 6.8	939	1.05 (0.94, 1.16)		1.05 (0.96, 1.16)		1.05 (0.96, 1.15)	
Triglycerides, mmol/L							
< 0.84	442	1.14 (1.01, 1.28)	0.052	1.00 (0.89, 1.12)	0.025	1.15 (1.03, 1.29)	0.624
0.84-1.22	684	1.11 (1.00, 1.24)		1.04 (0.94, 1.15)		1.06 (0.96, 1.18)	
≥ 1.22	799	1.00 (0.90, 1.12)		1.13 (1.03, 1.24)		1.06 (0.96, 1.17)	
Sex							
Female	578	1.02 (0.91, 1.14)	0.329	1.01 (0.89, 1.14)	0.309	1.03 (0.92, 1.14)	0.219
Male	1347	1.09 (1.00, 1.18)		1.08 (1.01, 1.16)		1.11 (1.03, 1.19)	
Smoking status							
Never	405	1.16 (1.02, 1.33)	0.003	1.01 (0.90, 1.15)	0.658	1.16 (1.03, 1.31)	0.420
Former	367	1.26 (1.09, 1.46)		1.09 (0.96, 1.24)		1.08 (0.95, 1.23)	
Current	1153	0.98 (0.90, 1.07)		1.08 (0.99, 1.16)		1.05 (0.97, 1.14)	
Diabetes status							
Other	1874	1.07 (1.00, 1.14)	0.702	1.07 (1.00, 1.14)	0.466	1.09 (1.02, 1.15)	0.672
Diabetes	51	0.99 (0.68, 1.44)		0.93 (0.64, 1.35)		0.98 (0.62, 1.56)	
**Overall**	1925	**1.07 (0.99, 1.14)**		**1.06 (1.00, 1.13)**		**1.08 (1.02, 1.15)**	

Continuous variables were categorized based on tertile cut-offs in controls. All models are adjusted for age, sex, period of recruitment, smoking status, body mass index, systolic blood pressure, history of diabetes at baseline, total cholesterol, and $\log_e$ triglycerides. *P values were calculated using likelihood ratio tests comparing models with and without interaction terms.
