# Supplementary material for: Hemostatic Factors and Risk of Coronary Heart Disease in General Populations: New Prospective Study and Updated Meta-Analyses
Source: PLoS One. 2013 Feb 7;8(2):e55175. doi: 10.1371/journal.pone.0055175 (PMC3567058; doi:10.1371/journal.pone.0055175)
Supplement: Figure S7 — Estimated within-person variability of t-PA antigen, D-dimer and VWF by time since baseline measurement in the published literature. Abbreviations: ARIC, Atherosclerosis Risk in Communities Study; BRHS, British Regional Heart Study; EAS, Edinburgh Artery Study; Fletcher, Fletcher Challenge Study; Reykjavik, Reykjavik Study. Each point represents unadjusted study- and time-specific estimates of within-person variability (eg, reported correlation coefficients or regression dilution ratios) in paired samples taken some time apart. Grey boxes represent unadjusted estimates. The relative sizes of the boxes are proportional to the inverse of the standard errors. (PDF) [file pone.0055175.s007.pdf]

Figure S7. Estimated within-person variability of t-PA antigen, D-dimer and VWF by time since baseline measurement in the published literature.

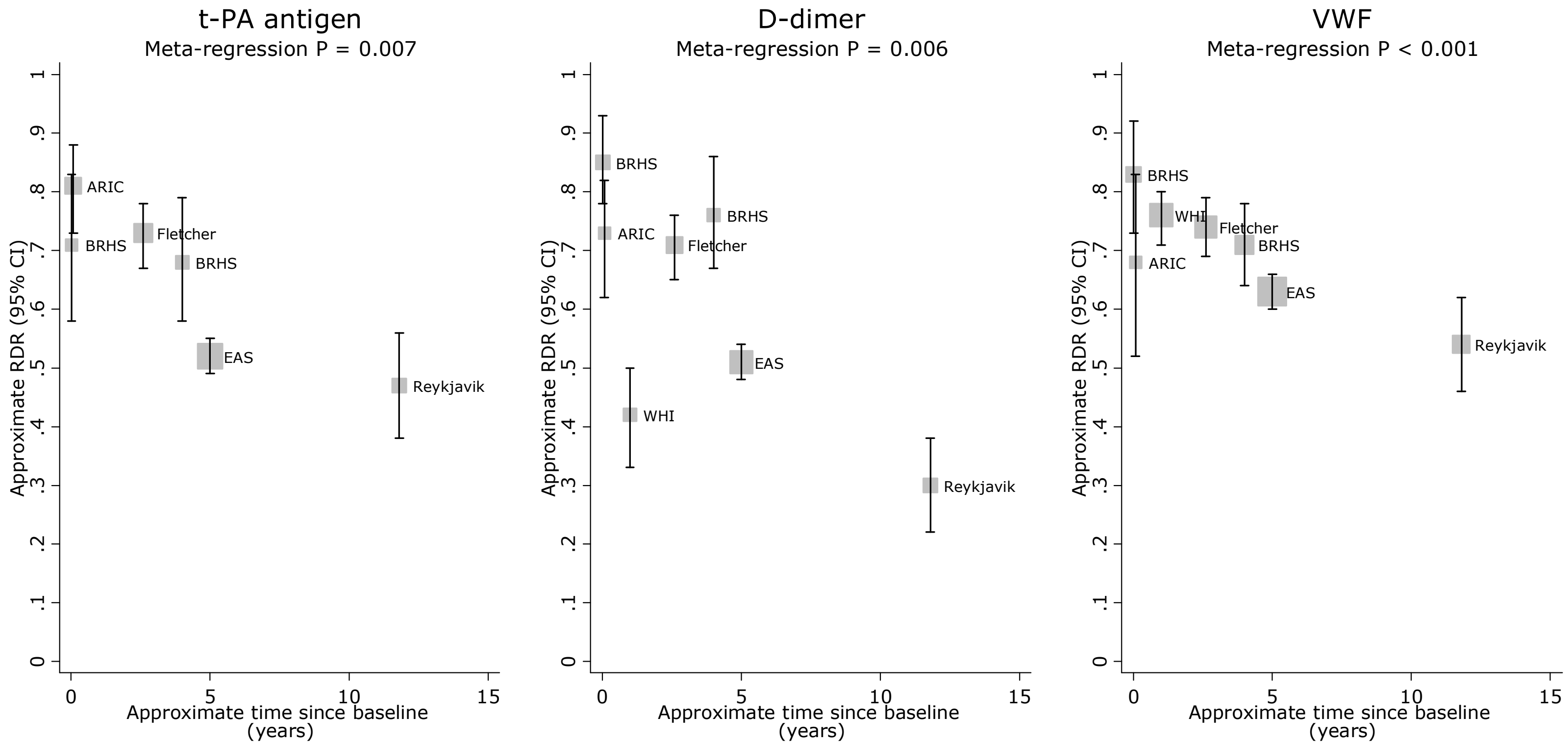

Abbreviations: **ARIC**, Atherosclerosis Risk in Communities Study; **BRHS**, British Regional Heart Study; **EAS**, Edinburgh Artery Study; **Fletcher**, Fletcher Challenge Study; **Reykjavik**, Reykjavik Study.
Each point represents unadjusted study- and time-specific estimates of within-person variability (eg, reported correlation coefficients or regression dilution ratios) in paired samples taken some time apart. Grey boxes represent unadjusted estimates. The relative sizes of the boxes are proportional to the inverse of the standard errors.
