# Supplementary material for: Hemostatic Factors and Risk of Coronary Heart Disease in General Populations: New Prospective Study and Updated Meta-Analyses
Source: PLoS One. 2013 Feb 7;8(2):e55175. doi: 10.1371/journal.pone.0055175 (PMC3567058; doi:10.1371/journal.pone.0055175)
Supplement: Figure S8 — Meta-analyses of reported associations of t-PA antigen, D-dimer and VWF with coronary heart disease risk in population-based prospective studies. Where studies reported more than one odds ratio, the least adjusted estimate was used. Study acronyms are explained in the legend of Table 3. Summary estimates were calculated using random effects models. *Degree of adjustment:+minimally adjusted (typically adjusted for age and sex only);++plus adjustment for at least one non-lipid marker;+++plus adjustment for at least one lipid marker;++++plus adjustment for at least one inflammatory marker. Where studies reported relative risks with more than one level of statistical adjustment, the least adjusted estimate was used (most adjusted estimates are reported in Figure 4). (PDF) [file pone.0055175.s008.pdf]

Figure S8. Meta-analyses of reported associations of t-PA antigen, D-dimer and VWF with coronary heart disease risk. Where studies reported more than one odds ratio, the least adjusted estimate was used.

Marker / Study	No. of cases	Degree of adjustment*
**t-PA antigen**		
Glostrup	133	++++
PHS	231	+
ARIC	326	+
Fletcher	225	+
WHI	304	++
EAS	248	+
BWHHS	198	+
NSHDS	467	+
BRHS 2	198	+
Caerphilly	316	+
BRHS 1	606	+
PROSPER	372	+
Reykjavik	1925	+
**Total**	**5549**	
**D-dimer**		
Three-City	199	+++
CHS	73	+
PRIME	162	+
Fletcher	220	+
PHS	296	+
ARIC	326	+
Speedwell	165	+
MESA	207	+
BWHHS	198	+
BRHS 2	198	+
NSHDS	458	+
EAS	248	+
WHI	359	++
Caerphilly	310	+
WOSCOPS	485	++
PROSPER	373	+
BRHS 1	630	+
Reykjavik	1925	+
**TOTAL**	**6832**	
**VWF**		
Three-City	199	+++
NPHS-I	84	+
PRIME	162	+
Fletcher	223	+
BWHHS	198	+
BRHS 2	198	+
EAS	248	+
NSHDS	469	+
WHI	359	++
Caerphilly	316	+
Rotterdam	473	+++
PROSPER	369	+
BRHS 1	625	+
ARIC	737	+
Reykjavik	1925	+
**TOTAL**	**6585**	

Relative risk (95% CI) for coronary heart disease per 1 SD higher baseline level

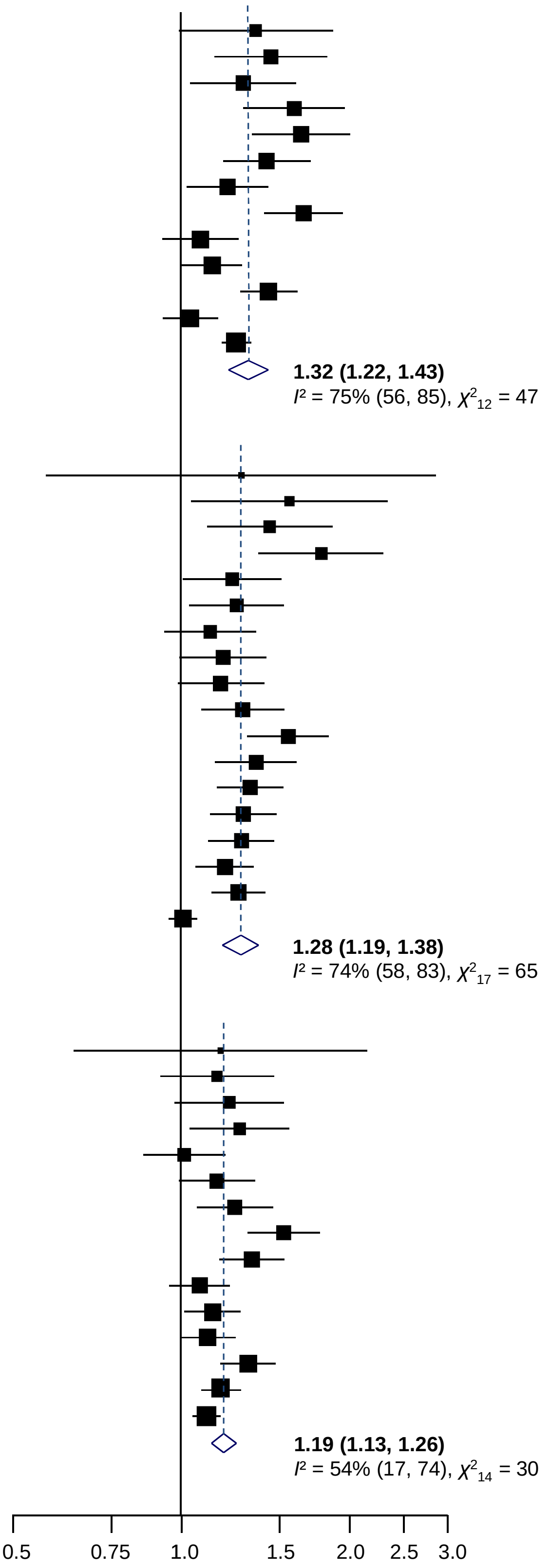

Study acronyms are explained in the legend of **Table 3**. Summary estimates were calculated using random effects models. *Degree of adjustment: + minimally adjusted (typically adjusted for age and sex only); ++ plus adjustment for at least one non-lipid marker; +++ plus adjustment for at least one lipid marker; ++++ plus adjustment for at least one inflammatory marker. Where studies reported relative risks with more than one level of statistical adjustment, the least adjusted estimate was used (most adjusted estimates are reported in **Figure 4**).
