# Supplementary material for: Hemostatic Factors and Risk of Coronary Heart Disease in General Populations: New Prospective Study and Updated Meta-Analyses
Source: PLoS One. 2013 Feb 7;8(2):e55175. doi: 10.1371/journal.pone.0055175 (PMC3567058; doi:10.1371/journal.pone.0055175)
Supplement: Figure S9 — Investigation of sources of possible heterogeneity in reported associations of t-PA antigen, D-dimer and VWF with coronary heart disease risk, according to various study-level characteristics. Summary estimates were calculated using random effects models. *P values are from meta-regression for differences in odds ratios across studies in different groups. (PDF) [file pone.0055175.s009.pdf]

**Figure S9.** Investigation of sources of possible heterogeneity in reported associations of t-PA antigen, D-dimer and VWF with coronary heart disease risk, according to various study-level characteristics.

| Subgroup | No. of studies | No. of cases | Relative risk (95% CI) for coronary heart disease per 1 SD higher baseline level | P value* |
|---|---|---|---|---|
| **t-PA antigen** | | | | |
| Study size | | | | |
| < 500 cases | 10 | 2591 | 1.15 (1.05, 1.27) | 0.633 |
| ≥ 500 cases | 3 | 2903 | 1.10 (1.00, 1.21) | |
| Sex | | | | |
| Men | 5 | 2698 | 1.11 (1.02, 1.19) | 0.847 |
| Women | 3 | 1056 | 1.15 (0.89, 1.47) | |
| Not reported separately | 6 | 1740 | 1.16 (1.03, 1.30) | |
| Duration of follow-up | | | | |
| < 10 years | 7 | 1799 | 1.10 (0.98, 1.23) | 0.367 |
| ≥ 10 years | 6 | 3695 | 1.17 (1.07, 1.28) | |
| Sample source | | | | |
| Plasma | 10 | 2830 | 1.12 (1.03, 1.23) | 0.656 |
| Serum | 3 | 2664 | 1.17 (1.02, 1.34) | |
| Storage temperature | | | | |
| > -70'C | 4 | 2912 | 1.20 (1.06, 1.36) | 0.321 |
| ≤ -70'C | 9 | 2582 | 1.10 (1.01, 1.20) | |
| Fasting status | | | | |
| Fasted | 10 | 4463 | 1.11 (1.03, 1.21) | 0.370 |
| Non-fasted | 3 | 1031 | 1.22 (1.10, 1.35) | |
| **Total** | **13** | **5494** | **1.13 (1.06, 1.21)** | |
| **D-dimer** | | | | |
| Study size | | | | |
| < 500 cases | 15 | 3871 | 1.25 (1.17, 1.34) | 0.375 |
| ≥ 500 cases | 3 | 2928 | 1.18 (1.03, 1.34) | |
| Sex | | | | |
| Men | 8 | 3593 | 1.18 (1.10, 1.27) | 0.341 |
| Women | 3 | 1135 | 1.17 (0.97, 1.40) | |
| Not reported separately | 8 | 2071 | 1.32 (1.17, 1.49) | |
| Duration of follow-up | | | | |
| < 10 years | 13 | 3228 | 1.25 (1.15, 1.35) | 0.615 |
| ≥ 10 years | 5 | 3571 | 1.21 (1.09, 1.35) | |
| Sample source | | | | |
| Plasma | 16 | 4244 | 1.25 (1.17, 1.33) | 0.387 |
| Serum | 2 | 2555 | 1.17 (0.96, 1.43) | |
| Storage temperature | | | | |
| >-70'C | 4 | 2968 | 1.15 (1.02, 1.30) | 0.163 |
| ≤ -70'C | 14 | 3831 | 1.26 (1.18, 1.35) | |
| Fasting status | | | | |
| Fasted | 14 | 5518 | 1.23 (1.14, 1.32) | 0.823 |
| Non-fasted | 4 | 1281 | 1.26 (1.05, 1.50) | |
| **Total** | **18** | **6799** | **1.23 (1.16, 1.32)** | |
| **VWF** | | | | |
| Study size | | | | |
| < 500 cases | 11 | 2900 | 1.16 (1.08, 1.26) | 0.979 |
| ≥ 500 cases | 4 | 3656 | 1.16 (1.07, 1.25) | |
| Sex | | | | |
| Men | 6 | 2732 | 1.16 (1.07, 1.25) | 0.219 |
| Women | 3 | 1135 | 1.05 (0.92, 1.19) | |
| Not reported separately | 7 | 2689 | 1.20 (1.11, 1.29) | |
| Duration of follow-up | | | | |
| < 10 years | 8 | 2152 | 1.12 (1.06, 1.19) | 0.344 |
| ≥ 10 years | 7 | 4404 | 1.19 (1.10, 1.30) | |
| Sample source | | | | |
| Plasma | 12 | 3922 | 1.15 (1.09, 1.23) | 0.808 |
| Serum | 3 | 2634 | 1.18 (1.02, 1.37) | |
| Storage temperature | | | | |
| > -70'C | 3 | 2798 | 1.19 (1.04, 1.37) | 0.650 |
| ≤ -70'C | 12 | 3758 | 1.15 (1.08, 1.22) | |
| Fasting status | | | | |
| Fasted | 13 | 5737 | 1.14 (1.08, 1.20) | 0.171 |
| Non-fasted | 2 | 819 | 1.30 (1.17, 1.45) | |
| **Total** | **15** | **6556** | **1.16 (1.10, 1.22)** | |

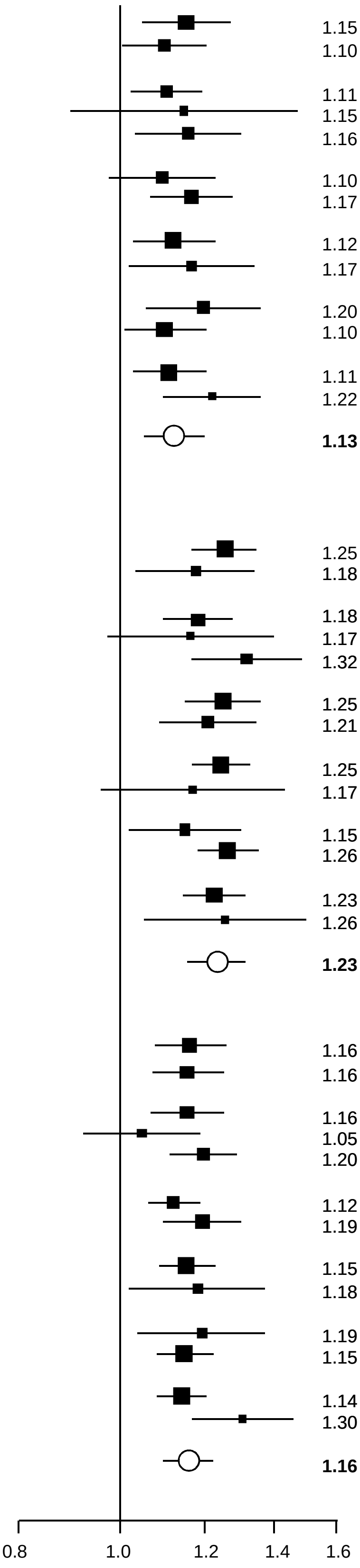


Summary estimates were calculated using random effects models. *P values are from meta-regression for differences in odds ratios across studies in different groups.
