# Supplementary material for: Hemostatic Factors and Risk of Coronary Heart Disease in General Populations: New Prospective Study and Updated Meta-Analyses
Source: PLoS One. 2013 Feb 7;8(2):e55175. doi: 10.1371/journal.pone.0055175 (PMC3567058; doi:10.1371/journal.pone.0055175)
Supplement: Figure S10 — Funnel plots of reported associations of t-PA antigen, D-dimer and VWF with coronary heart disease risk. The dotted lines show 95% confidence intervals around the overall summary estimate calculated using fixed effect models. Egger’s test for regression asymmetry: t-PA antigen, P = 0.086; D-dimer, P = 0.004; VWF, P = 0.290. (PDF) [file pone.0055175.s010.pdf]

Figure S10. Funnel plots of reported associations of t-PA antigen, D-dimer and VWF with coronary heart disease risk.

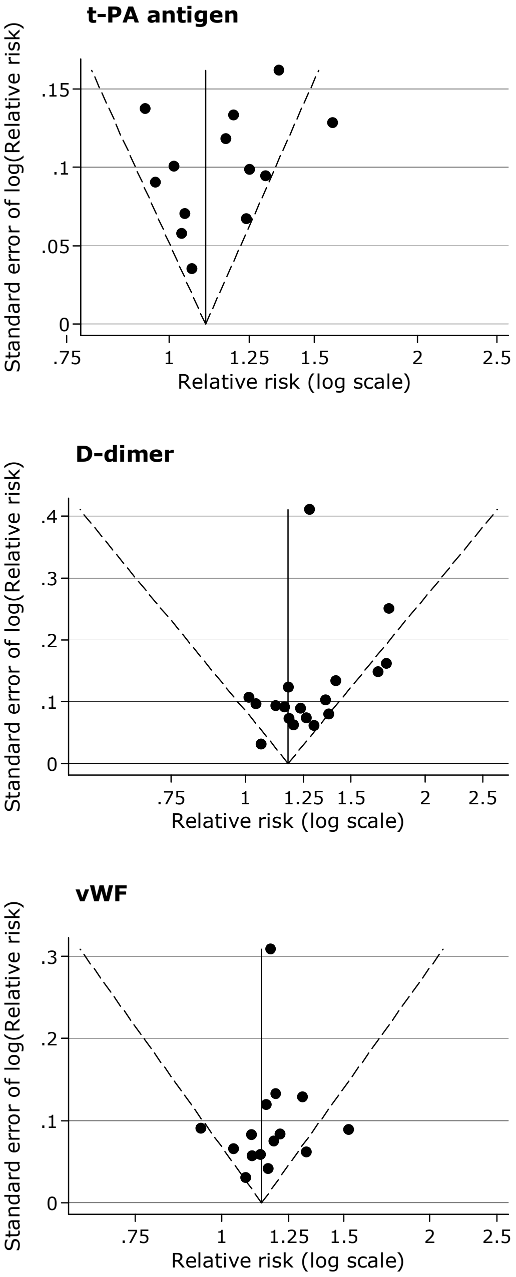

The dotted lines show 95% confidence intervals around the overall summary estimate calculated using fixed effect models. Egger's test for regression asymmetry: t-PA antigen, P = 0.086; D-dimer, P = 0.004; VWF, P = 0.290.
