# Supplementary material for: Hemostatic Factors and Risk of Coronary Heart Disease in General Populations: New Prospective Study and Updated Meta-Analyses
Source: PLoS One. 2013 Feb 7;8(2):e55175. doi: 10.1371/journal.pone.0055175 (PMC3567058; doi:10.1371/journal.pone.0055175)
Supplement: Table S1 — Comparison of baseline characteristics of coronary heart disease cases, randomly selected controls and the overall Reykjavik cohort. *Controls were matched to coronary heart disease cases by sex and age (in five-year age bands) and therefore differ from the remainder of the Reykjavik cohort. (PDF) [file pone.0055175.s011.pdf]

Table S1. Comparison of baseline characteristics of coronary heart disease cases, randomly selected controls and the overall Reykjavik cohort.

**Variable (Mean or %)**	**Nested case-control study**		**Overall Reykjavik cohort**
	**Coronary heart disease cases**	**Controls***	
	n=1925	n=3616	n=18912
Age, years*	54.2	55.2	52.8
Male, %*	70%	67%	48%
Current smoker, %	60%	48%	47%
Diabetes, %	3%	2%	2%
SBP, mmHg	146.7	141.9	138.3
BMI, kg/m^2	26.0	25.4	25.4
Total cholesterol, mmol/L	6.9	6.4	6.5

*Controls were matched to coronary heart disease cases by sex and age (in five-year age bands) and therefore differ from the remainder of the Reykjavik cohort.
