# Supplementary material for: Hemostatic Factors and Risk of Coronary Heart Disease in General Populations: New Prospective Study and Updated Meta-Analyses
Source: PLoS One. 2013 Feb 7;8(2):e55175. doi: 10.1371/journal.pone.0055175 (PMC3567058; doi:10.1371/journal.pone.0055175)
Supplement: Table S3 — Within-person variability of t-PA antigen, D-dimer and VWF plus other measured markers (mean time between repeated measurements, 11.6 years). (PDF) [file pone.0055175.s013.pdf]

Table S3. Within-person variability of t-PA antigen, D-dimer and VWF plus other measured markers (time between repeated measurements ~11.6 years).

	Number of paired measurements	Age- and sex-adjusted regression dilution ratio (95% CI)
**Physical measurements**		
Body mass index	369	0.91 (0.85, 0.97)
Systolic blood pressure	357	0.73 (0.64, 0.82)
**Lipid markers**		
Total cholesterol	370	0.61 (0.53, 0.68)
Log_e lipoprotein(a)	370	0.93 (0.86, 0.99)
**Inflammatory markers**		
Log_e interleukin 6	299	0.33 (0.23, 0.44)
Log_e C-reactive protein	368	0.57 (0.48, 0.66)
Log_e erythrocyte sedimentation rate	322	0.68 (0.57, 0.80)
**Hemostatic markers**		
Log_e t-PA antigen	370	0.47 (0.38, 0.56)
Log_e D-dimer	370	0.30 (0.22, 0.38)
Log_e VWF	371	0.55 (0.47, 0.63)
