# Supplementary material for: Hemostatic Factors and Risk of Coronary Heart Disease in General Populations: New Prospective Study and Updated Meta-Analyses
Source: PLoS One. 2013 Feb 7;8(2):e55175. doi: 10.1371/journal.pone.0055175 (PMC3567058; doi:10.1371/journal.pone.0055175)
Supplement: Methods S1 — Systematic review and updated meta-analysis. (PDF) [file pone.0055175.s014.pdf]

Methods S1

Systematic review and updated meta-analysis

We sought prospective cohort studies published before October 19th 2012 that (i) reported on associations of any of the markers t-PA antigen, D-dimer or VWF with coronary heart disease outcomes, (ii) had a minimum follow-up time of one year, and (iii) had been conducted in essentially general populations (i.e. participants that had not been recruited on the basis of pre-existing disease). Computer-based searches of 'PubMed', 'Scientific Citation Index Expanded', 'Conference Proceedings Citation Index', 'EMBASE' and 'Literatura Latino Americana em Ciências da Saúde' combined terms related to coronary heart disease, study design and to each of t-PA antigen, D-dimer and VWF without language restriction (**Figure S2**). Reference lists of articles identified (including review articles and meta-analyses) were scanned for additional relevant studies. In case of multiple publications, the most up-to-date or comprehensive information was extracted. We corresponded with study authors to obtain additional information if study results were described ambiguously or not reported in sufficient detail. Statistical tests were two-sided and used a significance level of $P < 0.05$. Results reported in this publication were presented in abstract form at the International Society on Fibrinolysis and Proteolysis meeting in 2010 [1].

References

1. Thompson A, Lowe G, Rumley A, Eiriksdottir G, Danesh J, et al. (2010) Tissue plasminogen activator, fibrin D-dimer and Von Willebrand factor antigens and risk of future coronary heart disease. J Thromb Haemost 8 Suppl 1: 23. Abstract.
